# Draft genomes of 3 cyanobacteria strains and 17 co-habiting proteobacteria assembled from metagenomes

**DOI:** 10.1128/MRA.00460-23

**Published:** 2023-11-09

**Authors:** Maximilian Berthold, Martin Albrecht, Douglas A. Campbell, Naaman M. Omar

**Affiliations:** 1 Department of Biology, Mount Allison University, Sackville, Canada; 2 Institute of Biological Sciences, University of Rostock, Rostock, Germany; University of Southern California, Los Angeles, California, USA

**Keywords:** cyanobacteria, proteobacteria, metagenomics

## Abstract

*Cyanobium* and *Synechococcus* are prominent, globally distributed cyanobacteria genera with ecological significance. Here, we report the genomes of the marine *Synechococcus* sp. CCMP836 and two strains of *Cyanobium* (CZS25K and CZS48M) along with the genomes of 17 co-occurring proteobacteria. These genomes will improve the strain-specific ecological positions.

## ANNOUNCEMENT

The global brackish microbiome is dominated by *Synechococcus* and *Cyanobium* (1) with more strains available than sequenced genomes. *Cyanobium* spp. CZS25K and CZS48M were isolated from the southern Baltic Sea in 2017 (surface water, Darss-Zingst lagoon system, a eutrophic lagoon system with a salinity range of 2 to 6 (2), 54.43°N, 12.68°E; see (3) for details on isolation) and obtained from the Applied Ecology Culture Collection at the University of Rostock. *Synechococcus* sp. CCMP836 (synonyms WH 8007, 838BG) was isolated from the surface waters of the Gulf of Mexico in 1980 (19.75°N, −92.41°W) (4) and obtained from NCMA Bigelow.

**Fig 1:**
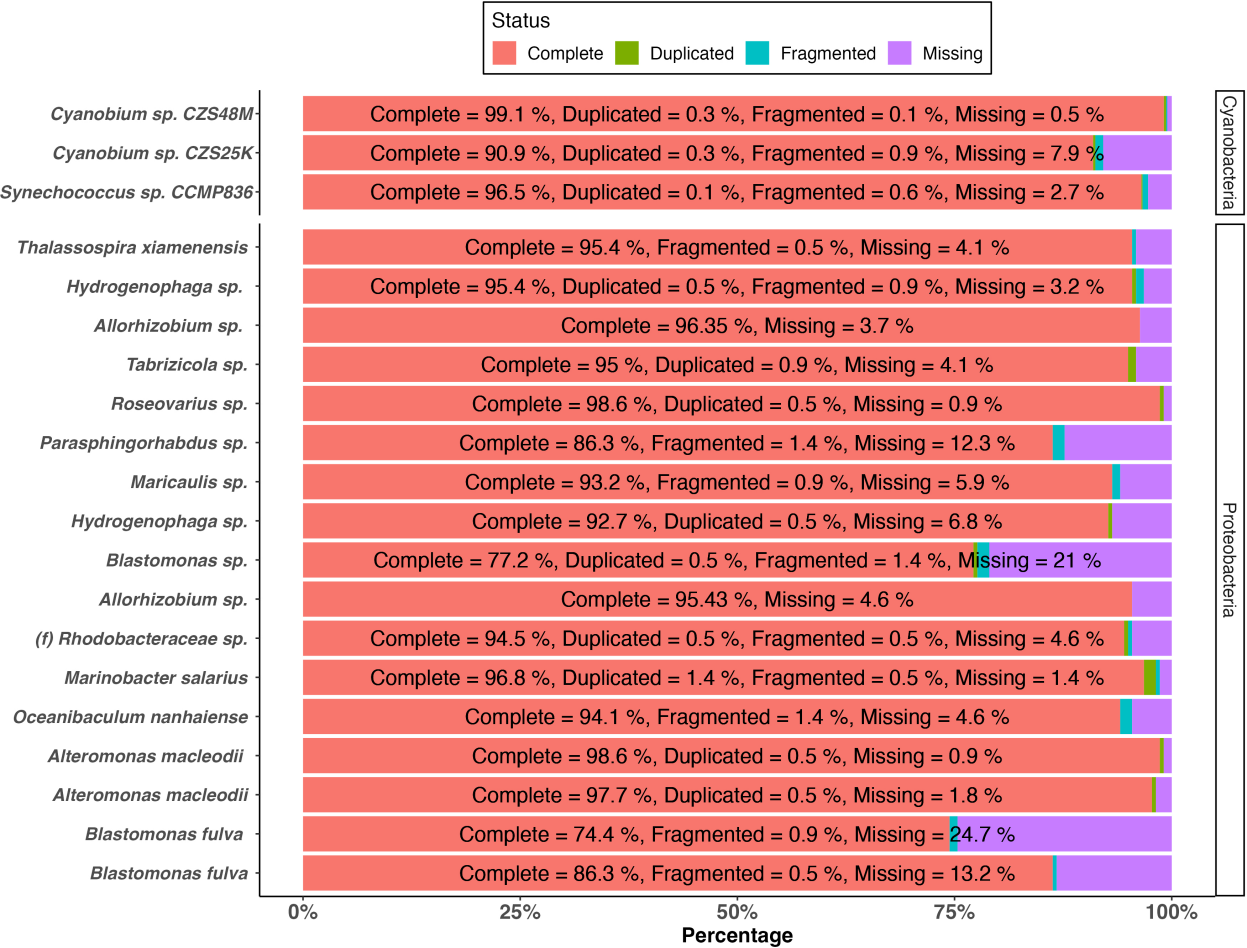
Predicted completeness of cyanobacterial and proteobacterial metagenome-assembled genomes based on core genes as analyzed by BUSCO.

CZS25K and CZS48M were grown in freshwater BG11 media (1 ppt salinity), whereas CCMP836 was grown in marine BG11 media (32 ppt salinity) under a 12:12 photoperiod on a shaker for 7 days before DNA extractions. DNA was extracted from xenic cultures of CCMP836, CZS25K, and CZS48M using a Qiagen DNeasy kit. Libraries were prepared using the NEBNext Ultra II DNA Library Prep kit for Illumina (New England Biolabs) as per the manufacturer’s recommendations and sequenced using the Illumina NovaSeq S4 lane using the Xp protocol as per the manufacturer’s recommendations by Genome Québec, Montréal, Canada, yielding 30.6 million, 28.4 million, and 32.4 million paired-end reads of 150 bp, respectively. In a second extraction, DNA was extracted from xenic cultures of CCMP836, CZS25K, and CZS48M using a Qiagen Genomic-tip 20 /G kit. Libraries were prepared using the Pacific Biosciences Preparing whole genome and metagenome libraries using the SMRTbell prep kit 3.0 protocol and sequenced using a PacBio Sequel II (Genome Québec, Montréal, Canada). No size selection was done prior to sequencing. The sequence quality was checked using FASTQC 0.11.9 (https://www.bioinformatics.babraham.ac.uk/projects/fastqc) and subsequently processed using Cutadapt 4.1 (5). Illumina reads and PacBio CCS reads were error-corrected and *de novo* assembled by Spades v3.15.4 (6) using the “-s” option for PacBio CCS reads and the “−1” and “−2” options for Illumina reads. Assemblies were aligned and sorted using Bowtie2 v2.4.4 (7) and Samtools v1.17 (8) and binned using MetaBAT2 (9) running on default parameters (Table 1). Samtools v1.17 (8) was also used to calculate genome coverage. Bin reliability was verified using FOCUS 1.5 (10) and CheckM v1.2.0 (11), and gaps were closed by GenomeFinisher (12) under default parameters using alternate Megahit v1.2.9 (13) and GenPipes (14) assemblies. Megahit v1.2.9 was run using the “-r” option for PacBio CCS reads and the “−1” and “−2” options for Illumina reads and the “meta-sensitive” preset. GenPipes was run by Genome Québec, Montréal, Canada, using PacBio reads. The completeness of bins was assessed using BUSCO v5.2.2 (15) (Figure 1) and subsequently annotated using PGAP (16). Taxonomy was assigned using the GTDB-Tk v2.1.1 classify workflow (17). Default parameters were used for all software unless otherwise specified.

**TABLE 1:** Taxonomy and attributes of binned metagenome-assembled genomes^*a*^

Phylum	Order	Organism	Total length (Mbp)	# contigs	Largest contig (bp)	GC (%)	*N*_50_	Genome coverage (×)	Source strain	Bin	Genome contamination (CheckM)	National Center for Biotechnology Information (NCBI) genome accession number	NCBI Sequence Read Archive (SRA) number
C	Synechococcales	*Cyanobium* sp. CZS48M	2.769251	37	457,164	66.93	98,032	3,538.57	CZS48M	9	0.14	JAUCZK000000000	SRR24524117 SRR24524116
C	Synechococcales	*Cyanobium* sp. CZS25K	3.100390	34	369,722	67.92	132,422	4,373.01	CZS25K	11	0.82	JAUCZB000000000	SRR24524118 SRR24524119
C	Synechococcales	*Synechococcus* sp. CCMP836	2.253937	19	357,230	63.53	155,493	1,145.01	CCMP836	24	0	JAUCYY000000000	SRR24524120 SRR24524121
P	Alteromonadales	*Alteromonas macleodii*	4.571015	58	300,811	44.65	130,570	37.377	CZS25K	4	0	JAUCZE000000000	SRR24524118 SRR24524119
P	Alteromonadales	*Alteromonas macleodii*	4.503556	49	286,734	44.67	138,090	324.57	CCMP836	21	0	JAUCYV000000000	SRR24524120 SRR24524121
P	Burkholderiales	*Hydrogenophaga* sp.	4.017909	52	251,823	66.32	102,357	115.426	CZS48M	2	0	JAUCZG000000000	SRR24524117 SRR24524116
P	Burkholderiales	*Hydrogenophaga* sp.	3.855627	10	1,132,111	64.5	446,382	180.227	CZS25K	9	0	JAUCZF000000000	SRR24524118 SRR24524119
P	Caulobacterales	*Maricaulis* sp.	2.986590	32	433,653	60.81	129,008	233.057	CCMP836	23	0	JAUCYX000000000	SRR24524120 SRR24524121
P	Oceanibaculales	*Oceanibaculum nanhaiense*	3.500712	17	570,757	65.29	317,371	189.412	CZS48M	7	0	JAUCZI000000000	SRR24524117 SRR24524116
P	Oceanospirillales	*Marinobacter salarius*	4.275244	62	365,476	57.32	135,686	61.0175	CCMP836	13	0	JAUCYT000000000	SRR24524120 SRR24524121
P	Rhizobiales	*Allorhizobium* sp.	4.479681	30	509,794	61.4	227,190	40.1371	CCMP836	11	0	JAUCYR000000000	SRR24524120 SRR24524121
P	Rhizobiales	*Allorhizobium* sp.	4.342418	21	823,773	61.46	352,591	199.246	CZS25K	3	0	JAUCZD000000000	SRR24524118 SRR24524119
P	Rhodobacterales	*Roseovarius* sp.	4.804078	18	743,804	66.05	328,058	400.304	CCMP836	15	0	JAUCYU000000000	SRR24524120 SRR24524121
P	Rhodobacterales	*Tabrizicola* sp.	3.616173	17	987,302	63.3	372,849	319.115	CZS48M	3	0	JAUCZH000000000	SRR24524117 SRR24524116
P	Rhodobacterales	*Rhodobacteraceae* sp.	3.599685	21	573,686	63.23	224,738	249.302	CCMP836	22	0	JAUCYW000000000	SRR24524120 SRR24524121
P	Rhodospirillales	*Thalassospira xiamenensis*	4.764832	23	970,535	54.71	264,246	254.064	CCMP836	9	0	JAUCYZ000000000	SRR24524120 SRR24524121
P	Sphingomonadales	*Blastomonas fulva*	3.805544	56	387,436	64.57	116,093	62.7709	CZS25K	10	0	JAUCZA000000000	SRR24524118 SRR24524119
P	Sphingomonadales	*Blastomonas* sp.	3.658526	58	237,960	64.04	112,763	92.1295	CZS25K	1	0	JAUCZC000000000	SRR24524118 SRR24524119
P	Sphingomonadales	*Blastomonas fulva*	3.463794	8	1,112,259	64.39	693,030	1031.03	CZS48M	8	0	JAUCZJ000000000	SRR24524117 SRR24524116
P	Sphingomonadales	*Parasphingorhabdus* sp.	3.127999	28	434,863	59.39	180,829	20.2539	CCMP836	12	0.84	JAUCYS000000000	SRR24524120 SRR24524121

^*a*^ The phylum column indicates whether organisms are cyanobacteria (C) or proteobacteria (P).

## Data Availability

This project has been deposited at the NCBI under BioProject accession number PRJNA956506. The raw sequence metagenomic reads can be located on the SRA under accession numbers SRR24524116 to SRR24524121. Genome assemblies have been deposited at DDBJ/ENA/GenBank under accession numbers JAUCYR000000000 to JAUCZK000000000.
